# Simultaneous Determination of 25 Ginsenosides by UPLC-HRMS via Quantitative Analysis of Multicomponents by Single Marker

**DOI:** 10.1155/2021/9986793

**Published:** 2021-07-01

**Authors:** Xiujuan Jia, Chenxing Hu, Xuepeng Zhu, Ye Yuan, Yifa Zhou

**Affiliations:** ^1^National Demonstration Center for Experimental Biology Education, Northeast Normal University, Changchun 130024, China; ^2^School of Life Sciences, Northeast Normal University, Changchun 130024, China

## Abstract

A method using UPLC-HRMS has been developed for a rapid, simultaneous qualitative and quantitative analysis of twenty-five ginsenosides. Chromatographic separation was achieved on a C_18_ analytical column with an elution gradient comprising 0.1% aqueous formate/acetonitrile as the mobile phase. HRMS detection acquired full mass data for quantification and fullms-ddms^2^ (i.e., data-dependent scan mode) yielded product ion spectra for identification. Furthermore, quantitative analysis of multiginsenosides by single marker (QAMS) was developed and validated using a relative correction factor. Under optimal conditions, we could simultaneously separate eight groups of isomers of the 25 ginsenosides. Good linearity was observed over the validated concentration range for each analyte (*r*^2^ > 0.9924), showing excellent sensitivity (LODs, 0.003–0.349 ng/mL) and lower limit quantification (LOQs, 0.015–1.163 ng/mL). The LC-MS external standard method (ESM) and QAMS were compared and successfully applied to analyze the ginsenoside content from *Panax ginseng* roots. Overall, our UPLC-HRMS/QAMS approach provides high precision, stability, and reproducibility and can be used for high-throughput analysis of complex ginsenosides and quantitative analysis of multiple components and quality control of traditional Chinese medicines (TCM).

## 1. Introduction

Ginseng (*Panax ginseng* C.A. Meyer) has been used as an herbal medication for centuries. Ginsenosides, the major pharmacologically active ingredient in ginseng, have a wide range of pharmacological and therapeutic properties, e.g., improving brain function, preventing cancer, enhancing immune responses, and exhibiting antiaging, antiobesity, and antidiabetic effects [1, 2]. Up to now, about 300 ginsenosides have been detected from ginseng [3]. However, it has remained problematic to quantify all ginsenosides, difficult to distinguish among ginsenosides with the same molecular weight, and hard to acquire sufficient numbers of ginsenoside standards for quantification of their chemical and physical properties.

Several methods have been developed to assess ginsenoside content in ginseng. Among these approaches, high-performance liquid chromatography (HPLC) and HPLC-MS are by far the most employed analytical methods [4–8]. However, HPLC-UV has always shown considerable baseline noise and relatively poor sensitivity due to the weak UV absorption of ginsenosides. Moreover, ginsenosides that have been reported were not well separated, leading to inaccurate quantitation. HPLC-MS is a powerful tool for determining ginsenosides in diverse ginseng extracts [9, 10]. For instance, thirteen ginsenosides were simultaneously and quantitatively determined by HPLC-MS [9]. Among the various LC approaches, ultra-performance LC (UPLC) has provided better separations over short periods of time and increased sample throughput and sensitivity [11]. Among different MS analyzers, Orbitrap has provided much better accuracy, precision, and greater mass resolution [12].

In our present study, we report a novel UPLC-Orbitrap-HRMS approach that has been validated for rapid, simultaneous qualitative/quantitative analysis of twenty-five ginsenosides. UPLC and HRMS methods were optimized to obtain excellent peak separations and maximum signal in the MS detector. Quantitative analysis of complex ginsenosides is difficult in the absence of reference standards. Therefore, a quantitative analysis of the multiginsenoside by single marker (QAMS) was developed and validated using a relative correction factor. The LC-MS external standard method (ESM) and QAMS were compared and successfully applied to analyzing ginsenoside content from *Panax ginseng* root. The present work has provided a sensitive and accurate approach for elucidating ginsenoside constituents that can also be used for multicomponent quality control of TCMs.

## 2. Materials and Methods

### 2.1. Chemicals and Reagents

HPLC-grade acetonitrile and methanol were purchased for Fisher Scientific (Waltham, MA); ultrapure water (18.25 MΩ/cm) was prepared by a Mili-Q water system (Millipore, Bedford, USA). Reference ginsenosides, including ginsenosides Rg1, Re, Rf, Rb1, Rg2, Rc, Rh1, Rb2, Rb3, F1, Rd, GXVII, nFe, CO, nFd, F2, G75, Rg3, PPT, Mc, CY, CMx, CK, Rh2, and PPD, were isolated or transformed from ginseng and purified by a series of chromatography procedures in our laboratory. The purity of the twenty-five standards was >95%.

### 2.2. Liquid Chromatography

Ultra-performance liquid chromatography (UPLC) was performed using an UPLC system (Dionex Ultimate 3000, Thermo Scientific, USA) equipped with a binary gradient pump, an autosampler, and a thermostatic column compartment. Chromatographic separation was achieved on a HyperSil GOLD C18 column (2.1 mm × 50 mm, 1.9 *μ*m) at a flow rate of 0.35 mL/min. The gradient elution system consisted of water/0.1% formic acid (A) and acetonitrile (B) using the following gradient program: 0–4 min, 23–30% B; 4–8 min, 30% B; 8.-8.5 min, 30–35% B; 8.5–12 min, 35% B; 12–12.5 min, 35–39% B; 12.5–16 min, 39% B; 16–17.5 min, 39–41% B; 17.5–20 min, 41–80% B; 20–21 min, 80–95% B; 21–21.5 min, 95–23% B; 21.5–25 min, 23% B. Column temperature was set at 30°C. The temperature of the autosampler was set at 15°C, and the injection volume was 5 uL.

### 2.3. Mass Spectrometry

The UPLC system coupled to a Q-Orbitrap HRMS (Thermo-Fisher, USA) with ESI operating in the negative ion mode and using full mass and fullms-ddms^2^ scan types. The UPLC-Q-Orbitrap-HRMS acquired full MS data for quantification over the scan range of m/z 100–1500. The MS resolution was set at 70,000 FWHM, and the AGC target and the maximum inject time were set at 1.0 *e*^6^ and 100 ms, respectively. The fullms-ddms^2^ (i.e., data-dependent scan mode) provided product ion spectra for identification using the mass inclusion list. The data-dependent- (dd-) ms^2^ resolution was set at 17500 FWHM, and the AGC target was set at 2.0 *e*^5^ with a maximum injection time of 100 ms. The top 5 most intense ions were selected to perform MS/MS acquisition. Precursor ions were filtered by the quadrupole, which operates at an isolation window of m/z 4 Da. The normalized collision energy (NCE) of each ginsenoside was optimized by injecting a working mix standard solution at a concentration of 1 ug/mL. UPLC-HRMS data were controlled using Thermo Xcalibur 4.3 software (Thermo Finnigan, San Jose, CA, USA). In addition, the spray voltage was set at 2.8 kV, the capillary temperature was 320°C, and the S-lens RF level was fixed at 50 to obtain the best experimental conditions. Nitrogen was used as the sheath gas with a flow rate set at 40, and the aux gas flow rate set at 10. The aux gas heater temp was 300°C to achieve the highest signal intensity.

### 2.4. Preparation of Calibration Standards

The standard stock solutions of ginsenoside Rg1, Re, Rf, Rb1, Rg2, Rc, Rh1, Rb2, Rb3, F1, Rd, GXVII, nFe, CO, nFd, F2, G75, Rg3, PPT, Mc, CY, CMx, CK, Rh2 and PPD were prepared by dissolution in methanol at a final concentration of 1.0 mg/mL. Appropriate aliquots of the 25 stock solutions were then mixed to prepare a final standard solution. The stock solution was diluted with methanol to achieve serial working solutions.

### 2.5. Ginseng Sample Solutions

Freeze-dried ginseng root powder (purchased from Ningbo Gianon Biotech Co., Ltd.) was dissolved in 1 mL methanol and filtered through a 0.22 *μ*m filter for further UPLC-Q-Orbitrap-MS analysis.

### 2.6. Method Validation

#### 2.6.1. Selectivity

The chromatogram of a blank methanol sample was compared with a mixed ginsenoside standard solution and sample solution to investigate whether the target analytes had the same retention time with other unrelated components present in the sample.

#### 2.6.2. Calibration Curve and Sensitivity

The mixed solution of 25 kinds of single standards with concentrations of 0.001, 0.002, 0.01, 0.02, 0.05, 0.1, 0.2, 0.5, and 1.0 ug/mL for mass spectrometer detector was injected in sextuplicate (*n* = 6). A standard regression line was drawn with the standard sample concentration (*x*) and peak area (*y*). The areas under analyte peaks of standards and noise around corresponding analyte peaks were measured by injection samples in sextuplicate (*n* = 6). The limit of detection (LOD) had a signal-to-noise ratio of 3 (S/N = 3), and the limit of quantitation (LOQ) had a signal-to-noise ratio of 10 (S/N = 10).

#### 2.6.3. Precision and Accuracy

0.2 ug/mL of mixed ginsenoside standard solutions was prepared and injected six consecutive times (*n* = 6). The precision obtained by calculating the relative standard deviation (RSD) (set to be less than ±15%) and the accuracy were expressed as a relative error (RE) between measured and targeted values required to be within ±15%.

#### 2.6.4. Stability

To determine sample stability, solutions were prepared for sequential injections at 0, 2, 6, 12, 24, and 48 h (*n* = 6). Specific peaks over accurate mass ranges were identified, and the area under each ginsenoside peak was recorded. The stability was expressed as RSD%.

#### 2.6.5. Reproducibility

The reproducibility of the method was assessed by preparing 0.2 ug/mL of mixed ginsenoside standard solutions in six replicates (*n* = 6). After injection, the exact peak with accurate mass range area of each ginsenoside was recorded, and the RSD% was calculated.

### 2.7. Calculation of Relative Response Factors of Ginsenosides

When a single reference was used to determine multiple components in samples, the concentration of each analyte (*Cx*) was calculated as the ratio between the peak area of the analyte in the sample solution (*Ax*) and the peak area of a chosen reference analyte in a standard solution as a unit concentration (*As/Cs*) and then calibrated by the relative response factor (RCF) of each analyte (*Fx*). The formula is as follows:

(1) $$\begin{array}{c} Cx=\frac{Ax}{As/Cs}\times Fx. \end{array}$$

The relative response factor (*Fx*) for each ginsenoside was calculated as the ratio of the peak area in a unit concentration between standards (*As/Cs*) and the analyte (*Ax/Cx*):

(2) $$\begin{array}{c} Fx=\frac{As/Cs}{Ax/Cx}. \end{array}$$

It is worth mentioning that the final value of RCF is the average value of multiple RCFs determined using a series of concentrations of the internal reference sample.

For comparison of this new QAMS approach with an ESM, the standard method difference (SMD) was calculated according to the following equations:

(3) $$\begin{array}{c} \text{SMD}=\frac{C_{\text{ESM}}-C_{\text{QAMS}}}{C_{\text{ESM}}}\times \;100\%, \end{array}$$

where *C*_ESM_ and *C*_QAMS_ are the concentrations of analytes assayed by the external standard and QAMS methods, respectively.

## 3. Results and Discussion

### 3.1. Optimization of UPLC Conditions

The separation of mixtures of ginsenosides by chromatography is very difficult. This is especially true for the simultaneous separation of eight groups of isomers (Rg1/Rf, Re/Rd/GXVII, Rh1/F1, Rc/Rb2/Rb3, nFe/CO/nFd, Rg2/G75/Rg3/F2, Mc/CY/CMx, and CK/Rh2). The use of an appropriate chromatographic column is a key factor for separating ginsenosides. Therefore, two chromatographic columns, including HyperSil GOLD C18 (2.1 mm × 100 mm, 1.9 *μ*m) and HyperSil GOLD C18 (2.1 mm × 50 mm, 1.9 *μ*m) columns, were employed and compared as shown in Figure S1 in the Supplementary Materials for comprehensive image analysis. Our results demonstrate that the HyperSil GOLD C18 (2.1 mm × 50 mm, 1.9 *μ*m) column not only exhibited better resolution and higher peak capacity but also could separate ginsenoside isomers Rg3 and G75. Therefore, the HyperSil GOLD C18 (2.1 mm × 50 mm, 1.9 *μ*m) column was chosen for this study.

To improve sensitivity and resolution, weak acid has usually been added to the water phase [13, 14]. Using acetonitrile as the organic phase, the effect of formic acid as an additive to the aqueous phase was investigated using the HyperSil GOLD C18 (2.1 mm × 50 mm, 1.9 *μ*m) column. Our results show that the addition of 0.1% aqueous formate/acetonitrile enhanced the intensity of mass signals and improved peak shape, thus providing the optimal choice (Figure S2 in the Supplementary Materials).

The effects of varying flow rate from 0.2 mL/min to 0.35 mL/min were assessed by observing the resolution of each analyte (Figure S3 in the Supplementary Materials). Increasing the flow rate significantly shortened analysis time and improved resolution. At an optimal flow rate of 0.35 mL/min, all isomeric compounds were effectively separated over a short period of time, except for ginsenosides Rg3 and G75. Finally, column temperatures of 30°C, 35°C, and 40°C were investigated. As shown in Figure S4 in Supplementary Materials, increasing the column temperature slightly influenced the retention time and resolution of each ginsenoside. In general, a temperature of 30°C was optimal due to a shorter retention time and better resolution for ginsenosides Rg3 and G75.

Overall, optimal chromatograms for the 25 analytes were obtained within 25 min and showed stable baselines and high resolutions.

### 3.2. Optimization of ESI-HRMS Conditions

In this study, ESI positive and negative modes were compared. All of the investigated ginsenosides exhibited strong [M-H]^−^ ions in the negative ion mode but comparatively low-abundance [M+H]^+^ ions and [M+Na]^+^ ions in the positive ion mode. Furthermore, the negative ion mode provided more direct structural information, consistent with previous studies [15, 16]. In order to rapidly acquire MS/MS fragmentation data for the identification of chemical constituents, the fullms-ddms^2^ data acquisition mode was used to simultaneously collect the information from both precursor ion and their related fragment ions in a single run. Mass parameters of the spray voltage (2.0–4.0 kV), the capillary temperature (250°C–350°C), and S-lens RF level (30–70) were optimized by infusing standard solutions to achieve maximum responses of precursor ions. Moreover, the sheath gas flow rate (20–40), aux gas flow rate (5–10), and aux gas heater temperature (250°C–350°C) were also optimized manually to achieve the greatest signal intensity. Furthermore, to make optimization easier, the stepped normalized collision energies (NCE) of (10 V, 15 V, and 20 V), (10 V, 20 V, and 35 V), and (20 V, 30 V, and 40 V) were used to obtain representative product ion spectra for each compound.

### 3.3. Method Validation

**Figure 1:**
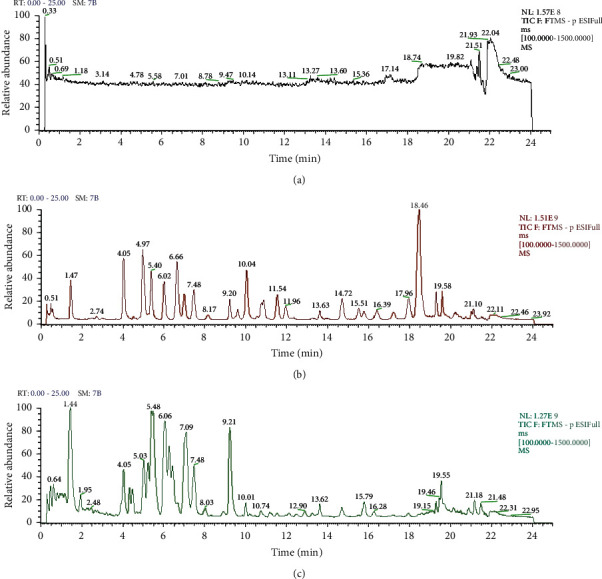
Total ion chromatograms. (a) Blank of methanol, (b) twenty-five ginsenoside standards, and (c) extract of *Panax ginseng* roots analyzed by UPLC-HRMS in negative ion mode.

To assess the selectivity of this method, chromatograms of a blank methanol sample, ginsenoside standards, and the ginseng sample itself are shown in Figure 1. All ginsenoside isomers were adequately separated on the column with elution times of 1.46 and 4.08 min for Rg1 and Rf (m/z 799), 1.46, 9.25, and 10.07 min for Re, Rd, and GXVII (m/z 945), 5.02 and 6.73 min for Rh1 and F1 (m/z 637), 6.09, 7.13, and 7.59 min for Rc, Rb2, and Rb3 (m/z 1077), 10.8, 11.61, and 12.01 min for nFe, CO, and nFd (m/z 915), 5.08, 13.68, 15.63, and 15.89 min for Rg2, F2, G75, and Rg3 (m/z 783), 17.29, 18.02, and 18.46 min for Mc, CY, and CMx (m/z 753), 19.31 and 19.6 min for CK and Rh2 (m/z 621), 14.75 min for PPT, and 20.99 min for PPD. There was no interference peak near the corresponding retention time of each target peak, which shows that this method has reasonably good specificity.

In addition, characteristics of calibration curves, including the range of linearity, the square of correlation coefficient (*r*^2^), limit of quantification (LOQ), and limit of detection (LOD) of each ginsenoside, are listed in Table 1.

All compounds showed excellent linearity over a relatively wide concentration range, with correlation coefficients (*r*^2^) of all calibration curves ranging from 0.9924 to 0.9998. The range of LOQs was within the range of 0.015–1.163 ng/mL. The range of LODs fell within the range of 0.003–0.349 ng/mL. In the stability test, analytes did not significantly degrade after storage of test solutions at room temperature for 48 h (RSD≤3.77%). Additionally, variation in RSD reproducibility was less than 3.86%. In terms of precision, RSDs ranged from 0.52% to 2.37%, and accuracy (RE%) ranged from 0.06% to 3.92%. These results are shown in Table 2, with all values being within an acceptable range.

### 3.4. Qualitative Analysis of Ginsenosides

Twenty-five authentic standards and ginsenoside mixture from *Panax ginseng* root were determined using UPLC-Orbitrap-HRMS under optimized conditions. Typical total ion chromatograms are shown in Figure 1. In the present study, the use of the fullms-ddms^2^ acquisition mode with scans ranging from 100 to 1500 m/z in negative ion mode combined with MS/MS fragmentation for the top 5 ions was proposed to aid in the structural identification of the components. The formula data, retention time, and experimental molecular mass and MS/MS fragment information are shown in Table 3. This provided abundant information that can be used as the basis for identifying constituents from *Panax ginseng* roots. Here, a total of 43 ginsenosides were identified. Among the 43 ginsenosides found, 25 ginsenosides (including peaks 3, 4, 5, 8, 9, 11, 12, 15, 17, 18, 21, 22, 23, 24, 25, 30, 31, 33, 34, 36, 37, 38, 39, 42, and 43) were identified as Rg1, Re, Rf, Rh1, Rg2, Rb1, Rc, F1, Rb2, Rb3, Rd, GXVII, nFe, CO, nFd, F2, PPT, G75, Rg3, Mc, CY, CMx, CK, Rh2, and PPD, by comparing retention times and high-resolution accurate mass and ion fragment data with the reference to available standard ginsenosides. In addition, the other 18 ginsenosides were tentatively identified using high-resolution accurate mass and ion fragments and the retention sequence by comparing values with those from the literature [3, 17–19].

**Figure 2:**
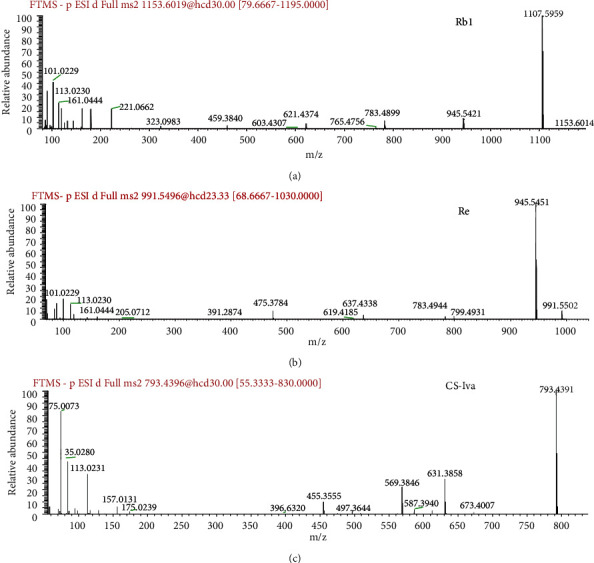
The MS/MS spectrum of ginsenoside-Rb1 (a), ginsenoside-Re (b), and ginsenoside-CS-Iva (c).

The production of adduct ions depends on the modifier added in the mobile phase [20, 21]. In this study, we added 0.1% formic acid in water that facilitated formation of the [M + COOH]^−^ solvent adduct ion. The [M-H]^−^ ion was generated from the [M + COOH]^−^ ion, following the loss of one HCOOH unit. The ginsenosides were grouped structurally as having one or more hydrophilic glycoside moieties combined with a lipophilic triterpene derivative. The glycosidic bond was easily broken and then the common fragmentation patterns of ginsenosides were simultaneously or successively lost as glycosidic units until the formation of [Aglycon-H]^−^ ions. The species and amount of sugar moieties were detected in MS/MS data, where mass differences of 162 Da and 146 Da suggested the presence of a *β*-D-glucose and *α*-L-rhamnose, respectively. The mass difference of 132 Da indicated the presence of a pentose (*β*-D-xylose or *α*-L-arabinose (pyranose or furanose)) fragment. Based on the structural characteristics of ginsenosides, PPTs and PPDs generated aglycon ions at *m/z* 475 and *m/z* 459, respectively. As shown in Table 3, there are nine PPT ginsenosides, including compounds 1, 2, 3, 4, 5, 7, 8, 9, and 15 and twenty-three PPDs ginsenosides such as compounds 6, 10, 11, 12, 13, 16, 17, 18, 19, 21, 22, 23, 24, 25, 30, 33, 34, 35, 36, 37, 38, 39, and 42. As an example, peak 11 (RT = 5.40), identified as ginsenoside Rb1, produced [protopanaxadiol-H]^−^ at *m/z* 459.3840 in the MS/MS spectrum by successive loss of four Glc-H_2_O (162 Da) groups. As another example, peak 4 (RT = 1.46), identified as ginsenoside Re, produced [protopanaxatriol-H]^−^ at *m/z* 475.3784 via successive elimination of one Rha, one Glc, and two Glc, respectively. Moreover, the OAs displayed an aglycone ion *m/z* 455 corresponding to [oleanolic acid-H]^−^. There are three OAs (e.g., compounds 14, 20, and 32). Peak 20 was identified as Chikusetsusaponin Iva by comparing retention times and ion fragments with those previously reported [22]. This peak displayed a [M-H]^−^ ion at m/z 793.4395. In the fullms-ddms^2^ experiment, two major fragment ions at m/z 631.3858 and 455.3555 were observed, indicating that the structure of saponins contains one glucose and one glucuronate group. The fragment ion at m/z at 455.3555 indicated the presence of an oleanolic acid aglycon moiety that lost all linked glycosidic units. These results are shown in Figure 2.

### 3.5. Quantitative Analysis of Ginsenosides by QAMS

#### 3.5.1. Relative Response Factors of Ginsenosides

Selecting a proper internal standard is vital for the accuracy of multicomponents using QAMS. Using Rb2 as an internal reference (1.00), the relative response factors (RCF) against ginsenoside Rb2 for all of the ginsenoside standards for the *m/z* signals of the exact peaks with accurate mass ranges in the full scan mode were established by using five different concentrations. The average RCF of each compound, calculated using Equation (2), can be seen in Table 4. It should be noted that the RSDs of the RCFs of all ginsenosides were less than 5.08%, suggesting that the RCFs obtained on the same instrument at different concentrations have good reproducibility.

#### 3.5.2. Reproducibility of RCFs and Relative Retention Times

The effects of using various column temperatures (30°C, 35°C, and 40°C) and flow rates (0.25, 0.3, and 0.35 mL/min) on RCF were investigated in order to evaluate the reproducibility of the QAMS approach. Results are shown in Table 5. RSDs measured under different conditions were all less than 5.97%, indicating that the RCF calculated by the established QAMS method has good reproducibility. When the content of analytes is determined by QAMS, the correct localization of the peaks is crucial. The relative retention time (RT_R_) between measured components and the internal reference (Rb2) was calculated here using the ratio of *t*_*Rk*_ to *t*_*Rs*_. RSDs of RT_R_ were calculated to evaluate the influence of column temperature (30°C, 35°C, and 40°C) and flow rate (0.25, 0.3, and 0.35 mL/min). Our results demonstrated that RSDs measured at different temperatures were less than 1.79%, and at different flow rates were less than 7.60%. This indicates that RT_R_ is reproducible at different temperatures when the flow rate is constant. Thus, RT_R_ can be used to accurately locate the chromatographic peak of each analyte.

#### 3.5.3. Quantitative Measurement of *Panax ginseng* Roots

In this study, a new UPLC-HRMS approach combined with the external standard method (ESM) and QAMS was developed to simultaneously determine 25 ginsenosides from *Panax ginseng* root, with calibration curve-based quantification provided in Table 6. The comparison of the two methods on the precision and accuracy is listed in Table S1 (in Supplementary Materials). The results indicated that the precision (RSD%) range of ESM and QAMS was 0.52%–2.37% and 0.45%–3.44%, respectively, and the accuracy (RE%) range was 0.06%–3.92% and 0.03%–5.01%, respectively. Both the ESM method and QAMS method had good precision and accuracy for the contents of 25 ginsenosides within the allowable error range. Using the ESM method, the amounts of Rg1, Re, Rf, Rh1, Rg2, and F1, which are all PPTs type, were calculated as 53.85, 151.28, 29.34, 1.39, 29.22, and 0.55 mg/g, respectively. The amounts of Rb1, Rc, Rb2, Rb3, Rd, GXVII, nFe, CO, nFd, F2, G75, Rg3, Mc, CY, CMx, CK, and Rh2, which are PPDs type, were detected as 324.15, 97.77, 112.03, 17.94, 97.66, 2.56, 3.92, 2.22, 1.75, 1.25, 0.11, 2.95, 0.91, 2.42, 0.38, 1.42, and 0.33 mg/g, respectively. The aglycon of PPT and PPD was detected as 1.03 and 0.47 mg/g, respectively. Among them, Re, Rb1, Rc, Rb2, and Rd had the highest content in *Panax ginseng* root. Moreover, the content of F1, G75, PPT, Mc, CMx, Rh2, and PPD was generally low.

At present, QAMS, as an alternative approach for the quality control of TCMs, has attracted increasing interest in terms of quality assessment and control of complex multicomponent systems [23, 24]. In this study, the content of 25 compounds was determined using the QAMS method. Using Rb2 as internal reference (1.00), RCFs of Rg1, Re, Rf, Rb1, Rg2, Rc, Rh1, Rc, F1, Rb3, Rd, GXVII, nFe, CO, nFd, F2, G75, Rg3, PPT, Mc, CY, CMx, CK, Rh2, and PPD were 0.31, 0.47, 0.49, 3.23, 0.43, 0.09, 0.92, 0.08, 0.98, 0.35, 0.24, 0.66, 0.63, 1.03, 0.10, 0.09, 0.14, 0.98, 0.34, 0.48, 0.25, 0.14, 0.07, and 6.71, respectively. To validate the feasibility of using QAMS for quantitative analysis of ginsenosides, the standard method difference (SMD) was established between the QAMS and ESM. As shown in Table 6, there were no significant differences between results from QAMS and ESM methods according to the SMD% falling between 0.1% and 5.45%, illustrating that the use of QAMS in our approach is reliable and accurate for detecting the concentration of each component.

## 4. Conclusions

In this study, we report an innovative approach based on UPLC-HRMS combined with QAMS and ESM for the simultaneous determination of multiple ginsenosides. By optimizing detection conditions, we can acquire for each ginsenoside within 25 min a high-resolution UPLC chromatogram with excellent sensitivity and stability. The QAMS method established using relative response factors shows minimal difference (SMDs below 5.45%) compared to the external standard method, implying that the UPLC-MS-QAMS approach can be used as a replacement for the external standard method when standard substances are absent. For these 25 different ginsenosides, data reproducibility was very good at low concentrations for MS detection. Furthermore, our approach is simple to use, highly sensitive and accurate, and applicable for all kinds of ginseng samples and for quality control of ginseng containing products.

## Figures and Tables

**Table 1:** Calibration curves, limit of quantification (LOQ), and limit of detection (LOD) of UPLC-MS methods for determination of 25 ginsenosides.

Ginsenoside	RT	Calibration curve	Test range (ug/mL)	*r* ^2^	LOQ (ng/mL)	LOD (ng/mL)
Rg1	1.46	*Y* = 64984*X* + 980287	0.02-2	0.9990	0.106	0.032
Re	1.46	*Y* = 41225.1*X* + 688583	0.02-2	0.9959	0.164	0.049
Rf	4.08	*Y* = 42146.9X − 44017.1	0.01-0.5	0.9994	0.116	0.035
Rh1	5.02	*Y* = 238413X − 153145	0.002-0.2	0.9994	0.025	0.007
Rb1	5.44	*Y* = 6240.95*X* + 85602.4	0.02-2	0.9964	1.163	0.349
Rg2	5.08	*Y* = 52298.5X − 612776	0.05-1	0.9924	0.012	0.003
Rc	6.09	*Y* = 22275X − 24796.1	0.01-1	0.9991	0.182	0.055
F1	6.73	*Y* = 292355X − 175557	0.002-0.2	0.9995	0.029	0.009
Rb2	7.13	*Y* = 20778.7X − 36514.3	0.01-1	0.9994	0.313	0.094
Rb3	7.59	*Y* = 20695.1*X* + 48835.8	0.05-2	0.9994	0.513	0.154
Rd	9.25	*Y* = 61029.8X − 558838	0.1-2	0.9989	0.015	0.005
G17	10.07	*Y* = 85248.5X − 57819.7	0.002-0.2	0.9998	0.217	0.065
nFe	10.8	*Y* = 29919.9X − 7299.02	0.002-0.2	0.9996	0.192	0.058
CO	11.61	*Y* = 31396.7X − 13591.7	0.001-0.2	0.9990	0.217	0.065
nFd	12.01	*Y* = 20835.8X − 25094.8	0.01-0.2	0.9959	0.833	0.250
F2	13.68	*Y* = 212189X − 115446	0.002-0.2	0.9998	0.029	0.009
G75	15.63	*Y* = 231218X − 99660.6	0.001-0.2	0.9991	0.042	0.013
Rg3	15.89	*Y* = 149379X − 82845.1	0.002-0.2	0.9993	0.154	0.046
PPT	14.75	*Y* = 27249.2X − 50845.3	0.002-0.05	0.9984	0.150	0.045
Mc	17.29	*Y* = 58309.3X − 14125.5	0.001-0.2	0.9995	0.294	0.088
CY	18.02	*Y* = 41233.6*X* + 36194	0.001-0.2	0.9993	0.175	0.053
CMx	18.46	*Y* = 80902.7X − 22662.5	0.002-0.2	0.9990	0.189	0.057
CK	19.31	*Y* = 140955*X* + 96724.4	0.001-0.2	0.9998	0.018	0.005
Rh2	19.6	*Y* = 316346X − 188172	0.002-0.2	0.9996	0.016	0.005
PPD	20.99	*Y* = 3099.89X − 4843.21	0.002-0.2	0.9990	0.535	0.160

**Table 2:** Instrument precision, accuracy, sample stability, and repeatability of the assay (*n* = 6).

Ginsenoside	Precision (RSD%)	Accuracy (RE%)	Stability (RSD%)	Repeatability (RSD%)
Rg1	0.88	0.06	1.05	0.92
Re	0.97	0.87	1.23	1.26
Rf	0.78	0.11	0.84	1.10
Rh1	0.63	0.25	0.68	1.41
Rb1	0.93	1.06	1.89	1.24
Rg2	0.52	3.92	1.71	1.45
Rc	1.76	2.13	1.56	1.58
F1	0.91	0.93	0.95	1.03
Rb2	1.38	2.24	1.77	1.14
Rb3	1.55	1.07	2.71	2.00
Rd	1.60	1.64	0.70	0.78
GXVII	0.79	0.31	2.14	2.01
nFe	1.93	0.22	3.77	3.31
CO	0.97	0.11	1.54	2.48
nFd	1.55	0.22	2.15	1.74
F2	0.74	1.71	0.65	1.34
G75	1.16	2.60	1.06	3.40
Rg3	1.85	3.65	0.71	1.58
PPT	1.63	0.36	1.32	3.86
Mc	1.10	0.73	1.84	2.15
CY	1.02	0.19	2.87	1.86
CMx	0.67	0.17	1.36	1.39
CK	0.85	2.07	1.76	1.79
Rh2	1.42	2.25	1.13	1.17
PPD	2.37	0.94	1.08	1.23

RSD, relative standard deviation, RE, relative error.

**Table 3:** Characterization of compounds in *Panax ginseng* roots using UPLC-HRMS.

No.	Identification	Formula	*T*_*R*_ (min)	[M + COOH]^−^ (m/z)	[M-H]^−^(m/z)	MS^2^ fragment ions (m/z)
1	20-Glc-ginsenoside Rf	C48H82O19	1.05	1007.5447	961.5384	799.4883[M-H-Glc]^–^637.4325[M-H-2Glc]^–^475.3785[M-H-3G lc]^–^
2	Notoginsenoside R1 (nR1)	C47H80O18	1.17	977.5344	931.5288	799.4872[M- H-Xyl]^–^637.4320[M-H-Xyl-Glc]^–^475.3792[M-H-Xyl-2Glc]^–^
3^*∗*^	Ginsenoside Rg1	C42H72O14	1.42	845.4910	799.4869	637.4324[M-H-Glc]^–^475.3803[M-H-2Glc]^–^
4^*∗*^	Ginsenoside Re	C48H82O18	1.46	991.5502	945.5451	799.4931[M-H-Rha]^–^783.4944[M-H-Glc]^–^637.4338[M-H-Glc-Rha]^–^475.3784[M-H-2Glc-Rha]^–^
5^*∗*^	Ginsenoside Rf	C42H72O14	4.05	845.4898	799.4855	637.4332[M-H-Glc]^–^475.3791[M-H-2Glc]^–^
6	Ginsenoside Ra3	C59H100O27	4.33	1285.6436	1239.6372	1107.6023[M-H-Xyl]^–^945.5419[M-H-Xyl-Glc]^–^783.4863[M-H-Xyl-2Glc]^–^621.4360[M-H-Xyl-3Glc]^–^
7	Ginsenoside F3/F5	C41H70O13	4.48	815.4810	769.4754	637.4333[M-H-Ara]^–^475.3789[M-H-Ara-Glc]^–^
8^*∗*^	Ginsenoside Rh1	C36H62O9	4.96	683.4381	637.4322	475.3778[M-H-Glc]^–^
9^*∗*^	Ginsenoside Rg2	C42H72O13	5.03	829.4963	783.4898	637.4306[M-H-Rha]^–^475.3807[M-H-Rha-Glc]^–^
10	Ginsenoside Ra2	C58H98O26	5.23	1255.6328	1209.6276	1077.5858[M-H-Xyl]^–^945.5414[M-H-Xyl-Ara (*f*)]^–^783.4915[M-H-Xyl-Ara (*f*)-Glc]^–^621.4380[M-H-Xyl-Ara (*f*)-2Glc ]^–^459.3847[M-H-Xyl-Ara (*f*)-3Glc ]^–^
11^*∗*^	Ginsenoside Rb1	C54H92O23	5.40	1153.6014	1107.5956	945.5421[M-H-Glc]^–^783.4899[M-H-2Glc]^–^621.4374[M-H-3Glc]^–^459.3840[M-H-4Glc]^–^
12^*∗*^	Ginsenoside Rc	C53H90O22	6.06	1123.5912	1077.5854	945.5413[M-H-Ara (*f*)]^–^783.4907[M-H-Ara (*f*)-Glc] ^–^621.4377[M-H-Ara (*f*)-2Glc]^–^459.3847[M-H-Ara (*f*)-3Glc]^–^
13	Ginsenoside Ra2-isomer	C58H98O26	6.27	1255.6329	1209.6266	1077.5870[M-H-Xyl]^–^945.5418[M-H-Xyl-Ara (*f*)]^–^783.4917[M-H-Xyl-Ara (*f*)-Glc]^–^459.3863[M-H-Xyl-Ara (*f*)-3Glc]^–^
14	Ginsenoside Ro	C48H76O19	6.44	-	955.4915	793.4398[M-H-Glc]^–^631.3837[M-H-2Glc]^–^455.3540[M-H-2Glc-GlcA]^–^
15^*∗*^	Ginsenoside F1	C36H62O9	6.64	683.4385	637.4288	475.3827[M-H-Glc]^–^
16	Ginsenoside Ra1	C58H98O26	6.68	1255.6334	1209.6271	1077.5790[M-H-Xyl]^–^783.4831[M-H-Xyl-Ara (*p*)-Glc]^–^621.4320[M-H-Xyl-Ara (*p*)-2Glc]^–^
17^*∗*^	Ginsenoside Rb2	C53H90O22	7.09	1123.5912	1077.5859	945.5415[M-H-Ara (*p*)]^–^783.4908[M-H-Ara (*p*)-Glc]^–^621.4387[M-H-Ara (*p*)-2Glc]^–^459.3847[M-H-Ara (*p*)-3Glc]^–^
18^*∗*^	Ginsenoside Rb3	C53H90O22	7.48	1123.5916	1077.5838	945.5406[M-H-Xyl]^–^783.4916[M-H-Xyl-Glc]^–^621.4372[M-H-Xyl-2Glc]^–^459.3855[M-H-Xyl-3Glc]^–^
19	Ginsenoside Ra1-isomer	C58H98O26	8.03	1255.6338	1209.6284	1077.5868[M-H-Xyl]^–^945.5466[M-H-Xyl-Ara (*p*)]^–^783.4907[M-H-Xyl-Ara (*p*)-Glc]^–^621.4337[M-H-Xyl-Ara (*p*)-2Glc]^–^459.3868[M-H-Xyl-Ara (*p*)-3Glc]^–^
20	Chikusetsusaponin Iva (CS-Iva)	C42H66O14	8.91	-	793.4395	631.3858[M-H-Glc]^–^455.3555[M-H-Glc-GlcA]^–^
21^*∗*^	Ginsenoside Rd	C48H82O18	9.21	991.5497	945.5438	783.4911[M-H-Glc]^–^621.4377[M-H-2Glc]^–^459.3850[M-H-3Glc]^–^
22^*∗*^	Gypenoside XVII	C48H82O18	10.01	991.5506	945.5432	783.4890[M-H-Glc]^–^621.4405[M-H-2Glc]^–^
23^*∗*^	Notoginsenoside Fe (nFe)	C47H80O17	10.74	961.5374	915.5348	783.4901[M-H-Ara (*f*)]^–^621.4399[M-H-Ara (*f*)-Glc]^–^459.3845[M-H-Ara (*f*)-2Glc]^–^
24^*∗*^	Compound O (CO)	C47H80O17	11.17	961.5401	915.5327	783.4915[M-H-Ara (*p*)]^–^621.4379[M-H-Ara (*p*)-Glc]^–^459.3847[M-H-Ara (*p*)-2Glc]^–^
25^*∗*^	NotoginsenosideFd (nFd)	C47H80O17	11.54	961.5401	915.5330	783.4908[M-H-Xyl]^–^621.4370[M-H-Xyl-Glc]^–^459.3854[M-H-Xyl-2Glc]^–^
26	Ginsenoside Rg6	C42H70O12	12.09	811.4872	765.4798	619.4215[M-H -Rha]^–^
27	Ginsenoside Rk3	C36H60O8	12.51	665.4287	619.3349	—
28	Ginsenoside F4	C42H70O12	12.90	811.4871	765.4786	619.4219[M-H-Rha]^–^457.3643[M-H-Rha-Glc]^–^
29	Ginsenoside Rh4	C36H60O8	13.24	665.4284	619.3344	—
30^*∗*^	Ginsenoside F2	C42H72O13	13.62	829.4974	783.4861	621.4400[M-H-Glc]^–^459.3816[M-H-2Glc]^–^
31^*∗*^	PPT	C30H52O4	14.69	521.3853	475.3792	—
32	Glycoside D3a	C42H66O14	14.70	-	793.4392	613.3735[M-H-Glc-H_2_O]^–^455.3517[M-H-Glc-GlcA]^–^
33^*∗*^	Gypenoside LXXV (G75)	C42H72O13	15.51	829.4975	783.4913	621.4393[M-H-Glc]^–^459.3844[M-H-2Glc]^–^
34^*∗*^	Ginsenoside Rg3	C42H72O13	15.79	829.4951	783.4912	621.4393[M-H-Glc]^–^459.3845[M-H-2Glc]^–^
35	20 (R)-Rg3	C42H72O13	16.28	829.4974	783.4905	621.4382[M-H-Glc]^–^459.3864[M-H-2Glc]^–^
36^*∗*^	Ginsenoside Mc	C41H70O12	17.31	799.4866	753.4793	621.4371[M-H-Ara (*f*)] ^–^459.3869[M-H-Ara (*f*)-Glc]^–^
37^*∗*^	Compound Y (CY)	C41H70O12	17.94	799.4868	753.4804	621.4374[M-H-Ara (*p*)]^–^459.3866[M-H-Ara (*p*)-Glc]^–^
38^*∗*^	Compound Mx (CMx)	C41H70O12	18.42	799.4869	753.4770	621.4406[M-H-Xyl]^–^459.3858[M-H-Xyl-Glc]^–^
39^*∗*^	Compound K (CK)	C36H62O8	19.30	667.4473	621.4392	459.3828[M-H-Glc]^–^
40	Ginsenoside Rk1	C42H70O12	19.46	811.4865	765.4803	603.4224[M-H-Glc]^–^
41	Ginsenoside Rg5	C42H70O12	19.55	811.4869	765.4843	603.4230[M-H-Glc]^–^
42^*∗*^	Ginsenoside Rh2	C53H90O22	19.59	667.4438	621.4372	459.3831[M-H-Glc]^–^
43^*∗*^	PPD	C30H52O3	20.99	505.3909	459.3843	—

^*∗*^Identified with a standard reference.

**Table 4:** Relative response factor (RCF) and relative standard deviation (RSD) values of 25 ginsenosides.

Ginsenoside	Concentration (ng/mL)					Mean	RSD (%)
	10	20	50	100	200		
Rb2	1.00	1.00	1.00	1.00	1.00	—	—
Rg1	0.32	0.30	0.31	0.30	0.30	0.31	2.92
Re	0.48	0.46	0.47	0.48	0.46	0.47	2.13
Rf	0.48	0.48	0.52	0.48	0.50	0.49	3.64
Rb1	3.45	3.25	3.05	3.18	3.22	3.23	4.48
Rg2	0.44	0.43	0.46	0.42	0.42	0.43	3.86
Rh1	0.09	0.09	0.09	0.09	0.08	0.09	5.08
Rc	0.93	0.88	0.94	0.94	0.92	0.92	2.70
F1	0.07	0.08	0.08	0.08	0.08	0.08	2.83
Rb3	0.97	0.97	0.98	0.99	0.98	0.98	0.86
Rd	0.36	0.34	0.35	0.34	0.34	0.35	2.59
GXVII	0.24	0.24	0.25	0.23	0.24	0.24	2.95
nFe	0.62	0.67	0.66	0.69	0.68	0.66	4.07
CO	0.60	0.62	0.63	0.66	0.65	0.63	3.78
nFd	1.05	1.00	1.05	0.99	1.05	1.03	2.95
F2	0.10	0.10	0.09	0.10	0.10	0.10	4.56
G75	0.09	0.09	0.09	0.08	0.09	0.09	5.08
Rg3	0.14	0.14	0.14	0.13	0.14	0.14	3.24
PPT	1.00	0.97	0.95	0.99	1.01	0.98	2.45
Mc	0.33	0.34	0.34	0.36	0.34	0.34	3.20
CY	0.47	0.48	0.50	0.49	0.47	0.48	2.71
CMx	0.24	0.25	0.25	0.26	0.25	0.25	2.83
CK	0.14	0.13	0.14	0.15	0.14	0.14	5.05
Rh2	0.06	0.07	0.07	0.07	0.07	0.07	3.24
PPD	6.48	6.95	6.67	6.98	6.45	6.71	3.75

**Table 5:** Effects of column temperature and flow rate upon relative response factor (RCF) and relative retention time (RT_R_).

	Effects of column temperature				Effects of flow rate			
	RCF		RT_*R*_		RCF		RT_*R*_	
Ginsenoside	Mean	RSD%	Mean	RSD%	Mean	RSD%	Mean	RSD%
Rg1	0.29	3.45	0.21	0.00	0.30	3.33	0.22	5.33
Re	0.47	2.44	0.21	0.00	0.49	2.37	0.22	4.55
Rf	0.48	1.21	0.56	1.79	0.48	1.19	0.58	1.00
Rb1	3.08	3.83	0.77	0.75	3.19	1.58	0.76	2.02
Rg2	0.34	4.45	0.70	1.43	0.35	1.63	0.72	0.81
Rh1	0.09	3.94	0.70	1.66	0.09	3.32	0.71	0.00
Rc	0.91	3.34	0.86	0.67	0.92	0.00	0.85	1.18
F1	0.08	0.00	0.95	0.61	0.08	0.00	0.96	1.04
Rb3	0.98	3.11	1.06	0.00	0.99	1.01	1.07	0.54
Rd	0.32	3.13	1.29	0.45	0.33	1.73	1.24	5.26
GXVII	0.25	4.00	1.41	0.41	0.25	2.28	1.36	4.80
nFe	0.67	1.49	1.52	0.38	0.68	0.85	1.46	4.12
CO	0.64	2.40	1.64	0.71	0.63	2.44	1.58	3.16
nFd	1.06	1.44	1.69	0.34	1.05	1.45	1.64	3.37
F2	0.10	5.97	1.92	0.52	0.10	0.00	1.95	6.48
G75	0.09	3.27	2.19	0.00	0.09	4.61	2.12	3.55
Rg3	0.13	4.56	2.21	0.90	0.13	0.00	2.16	3.50
PPT	1.03	2.03	2.05	1.49	1.02	1.96	1.96	5.61
Mc	0.34	2.94	2.43	0.24	0.35	1.67	2.33	4.54
CY	0.45	4.44	2.53	0.40	0.45	3.37	2.41	6.08
CMx	0.26	3.85	2.59	1.68	0.28	5.52	2.45	7.54
CK	0.11	5.41	2.69	1.14	0.10	5.59	2.53	7.50
Rh2	0.07	5.09	2.72	1.18	0.07	3.36	2.57	7.60
PPD	6.76	1.78	2.90	1.43	6.73	1.43	2.75	7.46

**Table 6:** Comparative experiment results of the external standard method (ESM) and quantitative analysis of multiginsenosides by single marker method (QAMS) (mg/g).

No.	Ginsenoside	ESM	QAMS	SMD%
1	Rg1	53.85	53.80	0.10
2	Re	151.28	143.83	4.92
3	Rf	29.34	29.47	0.46
4	Rb1	324.15	316.05	2.50
5	Rg2	29.22	30.21	3.38
6	Rh1	1.39	1.35	2.54
7	Rc	97.77	96.82	0.97
8	F1	0.55	0.57	2.52
9	Rb2	112.03	112.03	-
10	Rb3	17.94	17.88	0.38
11	Rd	97.66	98.03	0.38
12	GXVII	2.56	2.54	0.91
13	nFe	3.92	3.71	5.33
14	CO	2.22	2.20	0.52
15	nFd	1.75	1.76	0.50
16	F2	1.25	1.24	0.71
17	G75	0.11	0.10	5.45
18	Rg3	2.95	2.86	3.05
19	PPT	1.03	1.01	1.53
20	Mc	0.91	0.88	3.69
21	CY	2.42	2.42	0.21
22	CMx	0.38	0.37	1.40
23	CK	1.42	1.41	0.18
24	Rh2	0.33	0.32	4.12
25	PPD	0.47	0.46	2.09

## Data Availability

The data used to support findings in this study are included within the article.

## Supplementary Materials

Supplementary Materials The TIC of twenty-five ginsenoside standards by UPLC-HRMS with different kinds of the chromatographic column and different kinds of mobile phase and comparison of flow rates and column temperatures of ginsenoside detection in this article are available in the Supplementary Materials document. In other words, the comparison of the external standard method (ESM) and quantitative analysis of multiginsenosides by single marker method (QAMS) on the precision and accuracy is available in the Supplementary Materials document.
