# Supplementary material for: Simultaneous Determination of 25 Ginsenosides by UPLC-HRMS via Quantitative Analysis of Multicomponents by Single Marker
Source: Int J Anal Chem. 2021 Jul 1;2021:9986793. doi: 10.1155/2021/9986793 (PMC8266465; doi:10.1155/2021/9986793)
Supplement: Supplementary Materials — The TIC of twenty-five ginsenoside standards by UPLC-HRMS with different kinds of the chromatographic column and different kinds of mobile phase and comparison of flow rates and column temperatures of ginsenoside detection in this article are available in the Supplementary Materials document. In other words, the comparison of the external standard method (ESM) and quantitative analysis of multiginsenosides by single marker method (QAMS) on the precision and accuracy is available in the Supplementary Materials document. [file 9986793.f1.docx]

## Supplementary Materials

**Simultaneous Determination of 25 Ginsenosides by UPLC-HRMS via Quantitative Analysis of Multicomponents by Single Marker**

Xiujuan Jia,^1,2^ Chenxing Hu,^2^ Xuepeng Zhu,^2^ Ye Yuan^2^ and Yifa Zhou^2^

*^1^* National Demonstration Center for Experimental Biology Education, Northeast Normal University, Changchun 130024, China

*^2^* School of Life Sciences, Northeast Normal University, Changchun 130024, China

Correspondence should be addressed to Yifa Zhou; zhouyf383@nenu.edu.cn

**Legend of Figure**

**Supplemental Figure S1**: The TIC of twenty-five ginsenoside standards by UPLC-HRMS with different kinds of chromatographic column: (A) HyperSil GOLD C18 (2.1 mm×100mm, 1.9μm) column；(B) HyperSil GOLD C18 (2.1 mm×50mm, 1.9μm) column.

**Supplemental Figure S2**: The TIC of twenty-five ginsenoside standards by UPLC-HRMS with different kinds of mobile phase: water (A), 0.1% formic acid in water (B).

**Supplemental Figure S3**: The comparing of flow rate of ginsenoside detection (A: 0.25 mL/min, B: 0.3 mL/min, C: 0.35 mL/min).

**Supplemental Figure S4**: The comparing of column temperature of ginsenoside detection (A: 30 ℃, B: 35 ℃, C: 40 ℃).


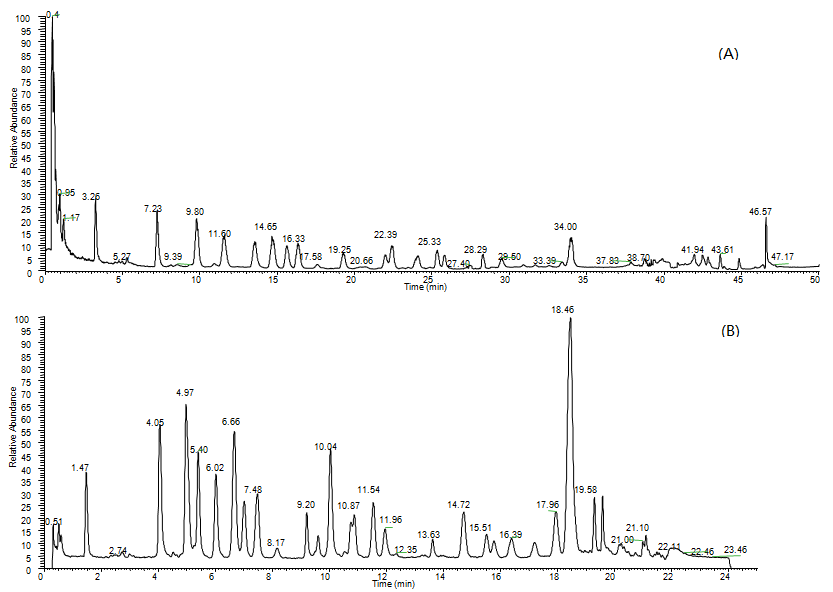


Figure S1：The TIC of twenty-five ginsenoside standards by UPLC-HRMS with different kinds of chromatographic column: (A) HyperSil GOLD C18 (2.1 mm×100mm, 1.9 μm) column；(B) HyperSil GOLD C18 (2.1 mm×50mm, 1.9 μm) column.


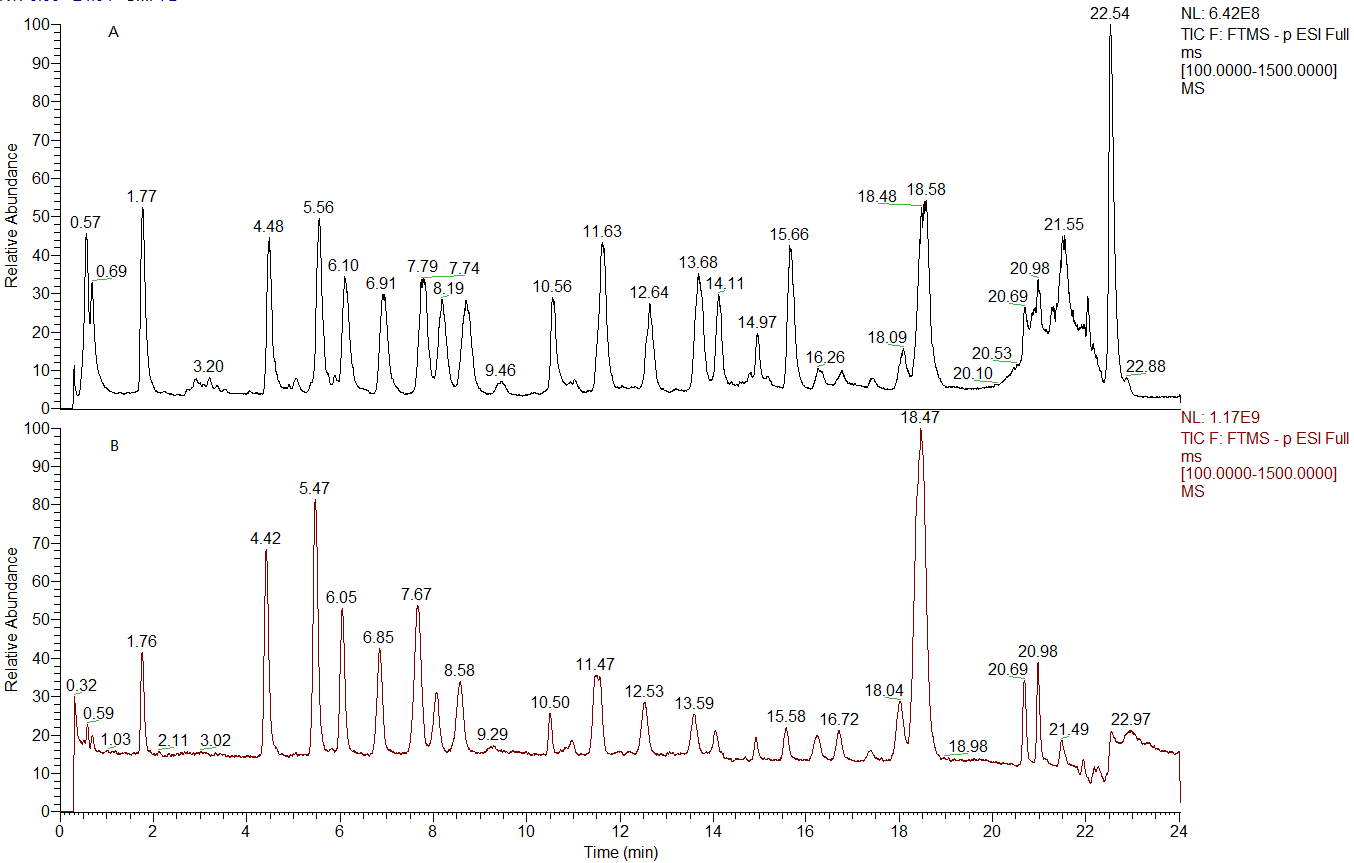


Figure S2：The TIC of twenty-five ginsenoside standards by UPLC-HRMS with different kinds of mobile phase: water (A), 0.1% formic acid in water (B).


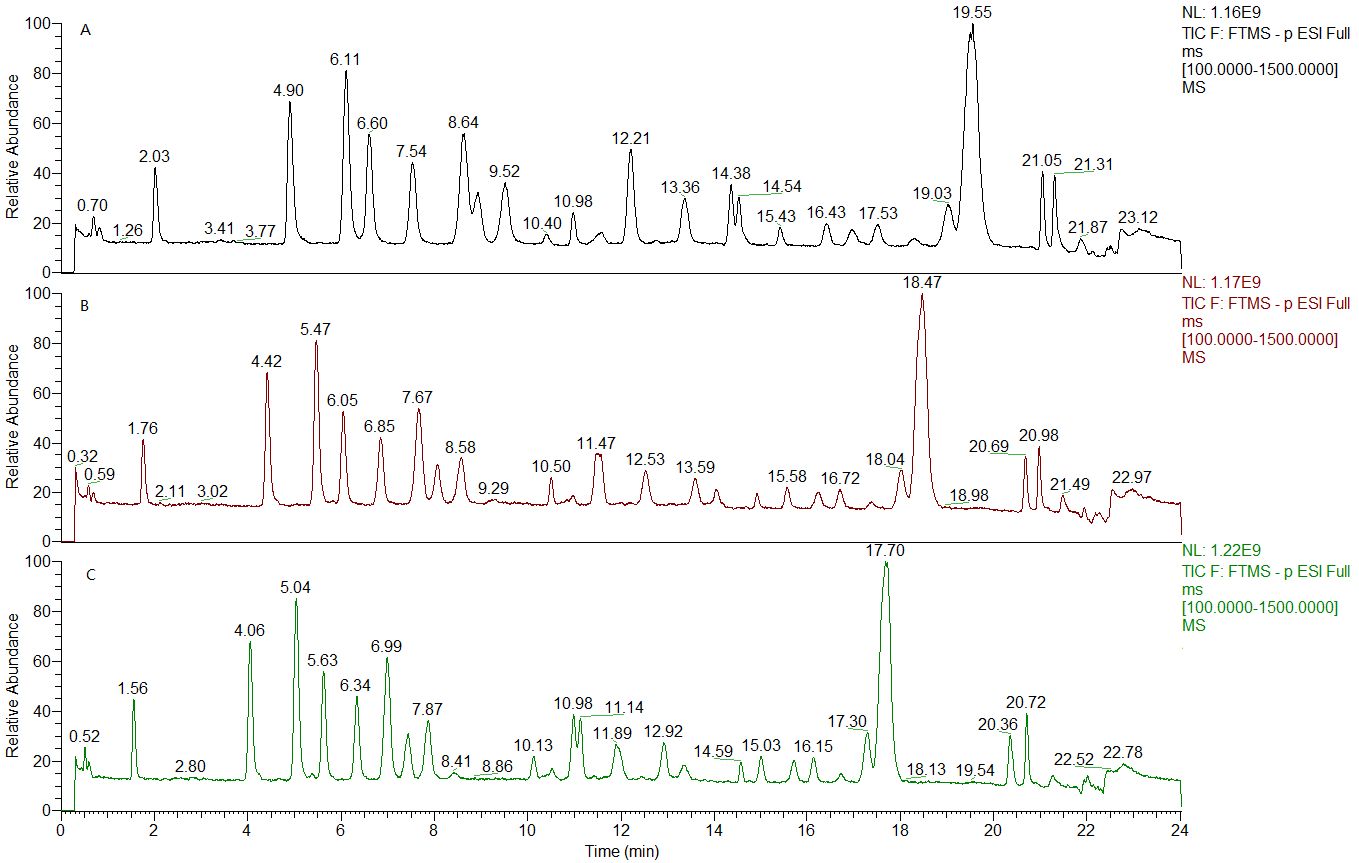


Figure S3: The comparing of flow rate of ginsenoside detection (A: 0.25 mL/min, B: 0.3 mL/min, C: 0.35 mL/min).


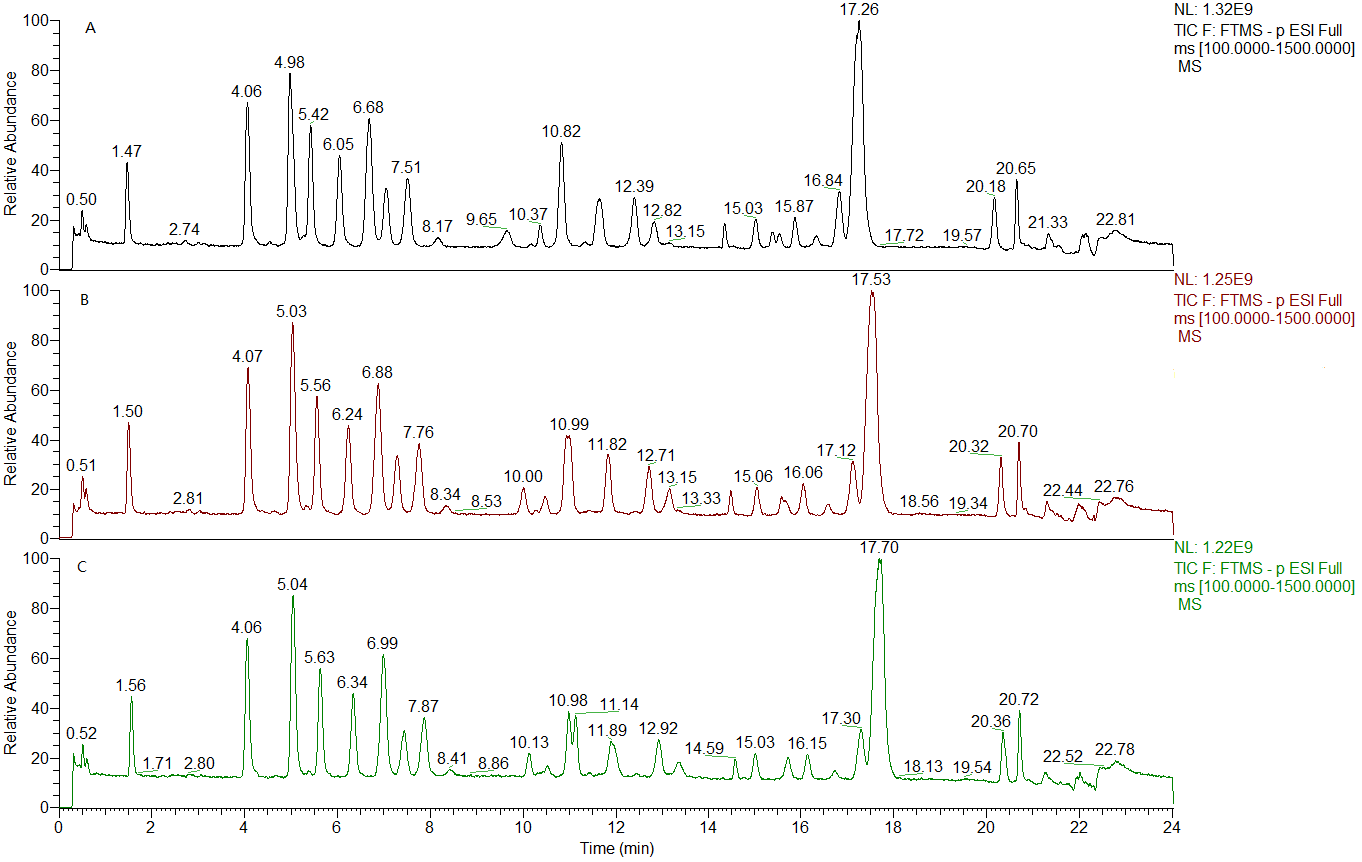


Figure S4: The comparing of column temperature of ginsenoside detection (A: 30 ℃, B: 35 ℃, C: 40 ℃).

Table S1: The comparison of the ESM and QAMS method on the precision and accuracy (*n=6*)

| Ginsenoside | Precision-ESM  (RSD%) | Precision-QAMS  (RSD%) | Accuracy-ESM  (RE%) | Accuracy-QAMS  (RE%) |
| --- | --- | --- | --- | --- |
| Rg1 | 0.88 | 0.88 | 0.06 | 0.27 |
| Re | 0.97 | 0.97 | 0.87 | 0.96 |
| Rf | 0.78 | 0.65 | 0.11 | 0.79 |
| Rh1 | 0.63 | 0.47 | 0.25 | 4.44 |
| Rb1 | 0.93 | 0.93 | 1.06 | 2.98 |
| Rg2 | 0.52 | 0.45 | 3.92 | 3.87 |
| Rc | 1.76 | 1.03 | 2.13 | 1.86 |
| F1 | 0.91 | 0.60 | 0.93 | 0.31 |
| Rb2 | 1.38 | 0.83 | 2.24 | 2.23 |
| Rb3 | 1.55 | 1.01 | 1.07 | 1.60 |
| Rd | 1.60 | 1.60 | 1.64 | 0.51 |
| GXVII | 0.79 | 0.58 | 0.31 | 0.03 |
| nFe | 1.93 | 2.79 | 0.22 | 4.96 |
| CO | 0.97 | 0.43 | 0.11 | 5.01 |
| nFd | 1.55 | 1.50 | 0.22 | 1.88 |
| F2 | 0.74 | 0.96 | 1.71 | 4.84 |
| G75 | 1.16 | 1.40 | 2.60 | 3.78 |
| Rg3 | 1.85 | 1.93 | 3.65 | 0.52 |
| PPT | 1.63 | 1.63 | 0.36 | 0.17 |
| Mc | 1.10 | 0.76 | 0.73 | 2.51 |
| CY | 1.02 | 1.25 | 0.19 | 3.32 |
| CMx | 0.67 | 0.98 | 0.17 | 2.37 |
| CK | 0.85 | 0.82 | 2.07 | 1.56 |
| Rh2 | 1.42 | 2.15 | 2.25 | 3.92 |
| PPD | 2.37 | 3.44 | 0.94 | 1.72 |
